# Dark or Short Nights: Differential Latitudinal Constraints in Nestling Provisioning Patterns of a Nocturnally Hunting Bird Species

**DOI:** 10.1371/journal.pone.0036932

**Published:** 2012-05-16

**Authors:** Markéta Zárybnická, Erkki Korpimäki, Michael Griesser

**Affiliations:** 1 Department of Ecology, Faculty of Environmental Sciences, Czech University of Life Sciences Prague, Prague, Czech Republic; 2 Section of Ecology, Department of Biology, University of Turku, Turku, Finland; 3 Department of Ecology, Swedish University of Agricultural Sciences, Uppsala, Sweden; 4 Department of Ecology and Evolution, University Bern, Bern, Switzerland; University of Jyväskylä, Finland

## Abstract

In diurnal bird species, individuals breeding at high latitudes have larger broods than at lower latitudes, which has been linked to differences in the daily time available for foraging. However, it remains unclear how latitude is linked with parental investment in nocturnal species. Here, we investigate nestling provisioning rates of male Tengmalm's owls in two populations at different latitudes (Czech Republic 50°N; Finland 63°N) with the help of cameras integrated into nest boxes. Clutch sizes were smaller in the Czech population (CZ: 5.1±0.1; FIN: 6.6±0.1), but given the higher nestling mortality in the Finnish population, the number of fledglings did not differ between the two populations (CZ: 3.5±0.3; FIN: 3.9±0.2). Nestling provisioning patterns varied within days, over the reproductive season and between the two sites. Males delivered most food at dusk and dawn, having peak delivery rates at sun angles of −11° to −15° at both sites, and males increased the prey delivery rates with higher nestling requirements. Given the longer nights during summer in the Czech Republic compared to Finland, Czech males only showed a small shift in their delivery peak during the night from −17° in April to −14° in July. In contrast, Finnish males shifted their peak of prey delivery from −11° in April to −1° in July. Consequently, Czech males had a longer hunting time per night around midsummer when feeding young (360 min) than Finnish males (270 min). This suggests that nocturnal owl species in northern populations are constrained by the short nights during the breeding season, which can limit the number of young they can raise. Moreover, owls in northern populations are additionally constrained through the unpredictable changes in food availability between years, and both these factors are likely to influence the reproductive investment between populations.

## Introduction

In many animals, parents provide dependent offspring with food to ensure proper development [1], [2]. Sufficient nutrition during development enhances short-term offspring survival and helps to avoid long-term costs that can arise through compensatory growth early in life [3], [4]. Within species, the reproductive investment often varies between populations. A well established pattern is the increase in avian clutch sizes with increasing latitude [5], [6], [7], although within some bird species individuals breeding in the middle of their distribution range have the largest clutch sizes [8].

Ashmole [9] hypothesized that the reproductive investment increases with latitude as a consequence of higher winter mortality, which reduces the number of competitors during the breeding season. While comparative studies confirmed this pattern [10], a field study suggested that a key factor responsible for within species differences in parental investment across latitudes is day length [11]. The energy expenditure of great tits (*Parus major*) indicates that parents breeding at low latitudes are time constrained, where the short daylight period limits the time available for foraging. In contrast, great tits breeding at high latitudes have more time available for foraging and thus can raise larger broods [11]. Consequently, we would expect to find the reverse pattern in nocturnal species. Individuals breeding at high latitude are likely to be more time constrained than individuals breeding at lower latitudes, affecting the reproductive decisions, but to our knowledge this hypothesis remains so far untested.

Based on this background, we investigate here the influence of night-length on feeding rates of male Tengmalm's owls in two populations: one in Central Europe (Czech Republic) and one in Northern Europe (Finland). As in other owls and diurnal raptors, the males provide nearly all food to the females from egg laying onwards, and later on as well for the young until independence [12], [13], [14]. Tengmalm's owl is a cavity breeding species that usually raises one brood per year but exceptionally two [15], [16] and occurs throughout the Palaearctic and Nearctic. The breeding season of Tengmalm's owls starts between early March and late April [17], and fledglings leave the nest between May and July. The night length during this period differs substantially throughout the range of Tengmalm's owls and can vary by up to four hours between Central and North Europe (Table 1).

**Table 1:** Night length (time between sunset and sunrise) at the two sites during the breeding season and basic breeding data (mean ± SE) of the Finnish and Czech populations of Tengmalm's owls.

	nest type	Finland	number of nests	Czech Republic	number of nests	df; ddf	F	p-vale
Night length1^st^ April		10 h 27 min		11 h 06 min				
Night length 15^st^ May		5 h 54 min		8 h 31 min				
Night length 30^st^ June		3 h 50 min		7 h 35 min				
Egg laying date	all nests	4 April±1.5 days	64	28 April±3.7 days	39	1; 101	62.02	<0.0001
	camera nests	7 April±6.7 days	9	27 April±6.7 days	12	1; 18	4.34	0.05
Fledging date	all nests	2 June±1.6 days	55	28 June±4.7 days	24	1; 76	38.46	<0.0001
	camera nests	9 June±6.8 days	9	29 June±7.0 days	12	1; 18	4.03	0.06
Clutch size	all nests	6.6±0.1	70	5.1±0.1	35	1; 102	9.23	0.003
	camera nests	6.7±0.2	9	5.3±0.3	12	1; 18	18.32	0.0005
No. of nestlings	all nests	6.0±0.2	65	4.4±0.2	27	1; 89	8.35	0.004
	camera nests	6.3±0.2	9	4.6±0.3	12	1; 18	2.93	0.10
No. of fledglings	all nests	3.9±0.2	55	3.5±0.3	24	1; 76	0.11	0.75
	camera nests	3.6±0.6	9	3.8±0.4	12	1; 18	0.05	0.82
Brood reduction	all nests	2.3±0.2	55	0.9±0.3	24	1; 76	15.42	0.0002
	camera nests	2.8±0.6	9	0.8±0.3	12	1; 18	10.35	0.005

The statistical comparisons are done with General Linear Mixed Models (Normal distribution, identity-link) (date egg laying, fledging date), or with a Generalised Linear Mixed Model (Poisson distribution, log-link) (clutch size, number of nestlings, number of fledglings, brood reduction). All models included year as random factor. Brood reduction was calculated as the number of nestlings which disappeared from a nest box before fledgling date (ring or dead nestling found in nest box).

We first examine the availability of main prey species at the two study sites given that prey availability affects prey delivery rates in Tengmalm's owls [18]. Then we compare the basic breeding data at both sites during the years of this study. After having established these baseline data, we test the following three hypotheses: (i) Prey delivery rates vary throughout a day. Owls refrain from foraging at daytime [19] and males are predicted to deliver most prey at night. Within nights, males will have the highest prey delivery rates at dusk and dawn, given the limitation of the retinal capacity of vertebrate eyes at low light levels [20]. This constraint should limit especially the foraging behaviour of males in the Czech population, where nights are much darker than in Finland. (ii) Males will increase prey delivery rates with increased energetic demand of the brood (older nestlings, larger brood). (iii) Given the shorter and lighter nights in the Finnish population in particularly around midsummer, males could either increase their prey delivery rates, or expand their hunting period into more light parts of the night to overcome this constraint.

## Methods

Data for this study was collected in the Czech Republic (50°N, 13°E) (2004, 2006) and in Finland (63°N, 23°E) (2005). The Czech site (70 km^2^) is located in the Ore Mountains (730–960 m a.s.l.), close to the border to Saxony. The habitat at this study site is characterised by open areas and forest fragments. In open areas and on clear-cuts the vegetation is dominated by wood reeds (*Calamagrostis villosa*) and solitary trees (mostly European beech *Fagus sylvatica*). Secondary growth areas are dominated by prickly spruce *Picea pungens*, birch *Betula* spp., European mountain ash *Sorbus aucuparia* and European larch *Larix decidua*. Within the study site, 120 nest boxes for Tengmalm's owls were placed from 1999 onwards and the breeding population has been followed since then [21]. The Finnish study site is located in mid-western Finland in the Kauhava region, (50–110 m a.s.l.), covering about 1300 km^2^ [17], [22]. Here, the landscape is characterised by a mosaic of boreal forests which are commercially used, agricultural fields and peat-land bogs. In this population, Tengmalm's owls have been studied from 1973 onwards in nest boxes (N = 420 during 1983–1987, N = 470 from 1988 onwards) and natural cavities (N = 30) [23]. Given the higher elevation of the Czech site compared to the Finnish site, mean temperatures during the breeding season do not differ much, while the Czech site received more precipitation and had consequently deeper snow cover (Appendix S1).

### Assessment of prey availability

In Tengmalm's owls, the onset of the breeding season, clutch size and breeding success depends on the availability of main prey (voles and mice) [14], [22]. The population density of voles at the Czech site shows low peaks every 4 to 5 years (mean ± SE trapped voles = 1.1±0.2; min-max = 0–3.6) while mice populations peaks at irregular intervals during beech mast years (1.1±0.3; min-max = 0–5.2) [24]). In 2004, spring prey availability was higher than in 2006 (mean trapped voles and mice 2004: 7.3; 2006: 0.3). The relative amplitude of the population cycles are much more pronounced in Finnish study site and fluctuate in 3-year cycles (mean ± SE trapped voles = 6.9±1.7; min-max = 0.2–29.0 [17]). In poor vole years, the reproductive period of owls starts approximately one month later and the clutch sizes are smaller than in good vole years [17]. In 2005, the year used to study prey delivery rates in the Finnish population, prey availability was intermediate throughout the whole breeding season (mean trapped voles: 10.6). We therefore included the mean number of trapped small mammals in our analyses to control for year specific differences in prey availability.

We assessed the availability of small mammals using snap-traps in both study populations. The trapping was carried out in both study populations in late spring (beginning of June in the Czech site, mid May in the Finnish site) by setting up snap traps in squares (squares 100*100 m with 10 m spacing). The traps were left out for 3 days and checked daily. In the Czech study site, the total trapping effort was 1089 trap nights in both 2004 and 2006 (N = 3 locations), with a distance of 1.6 to 1.8 km between the trapping locations. In the Finnish study site, the total trapping effort was 1230 trap nights (N = 8 locations), with a distance of 2.3 to 15 km between the trapping locations. For each trapping site we calculated the number of captured individuals per 100 traps nights. All captured mammals (N = 83 in the Czech population, N = 166 individuals in the Finnish population) were identified to the species level.

### Basic breeding data (all nests)

We collected basic breeding data in both places by inspecting all nest boxes during the onset of the breeding period [17], [21], [22]. Occupied nest boxes were re-visited 1–3 times per week to count the number of eggs, hatchlings and fledglings, to trap male and female parents, and to ring nestlings (Table 1). Nest boxes which were empty during the first visit were revisited about one month after the initial check to locate nests of pairs with a late onset of reproduction. Nests which did not produce at least one fledgling were classified as brood failure. The number of fledglings was determined by checking the nest-boxes for starved young. Starved young were either found dead in the nest box during the breeding season, or if cannibalised by their siblings or parents, they could be identified with the help of their numbered metal ring which was left behind in the nest box.

### Camera nests

We studied nestling provisioning rates in 12 nests in the Czech study site (6 nests in 2004, 6 nests in 2006) and in 9 nests in the Finnish study site during the breeding season (Table 1). The nests were monitored continuously during 24 hours with the help of a camera system integrated into the lid of a nest box [25]. For the camera boxes, we chose randomly among suitable nest boxes (i.e. boxes where it was possible to remove the old box, appropriate timing, avoiding boxes located close to roads and paths to avoid the attention of members of the public to the camera boxes). Upon finding a suitable nest, we replaced the box with a camera box, and given that we had three camera boxes, we could monitor three nests simultaneously. Each time an owl entered the nest box, the infrared motion detector triggered the camera, taking digital images of the owl in the nest box entrance (N = 1–3 pictures). The infrared diodes light up under dark conditions to allow the identification of the prey that the owl delivered to the nest. To know which individual entered or left the nest box, all adults (before mounting the camera box) and young (at day 14–21) were fitted with a pit-tag ring (BR chip ring, BENZING). The pit-tag reader was integrated into the nest box front and registered all the movements of the marked individuals in and out of the box.

All the nests monitored with cameras were successful and at least one young fledged. We recorded activity at the nests during nestling and fledgling phase over similar periods (25.8±2.2 days (± SE) in the Czech population; 22.9±2.4 days in the Finnish population; t = 1.0, p = 0.3), starting with the recordings on 26 April in the Czech population and on 19 April in the Finnish population. On average 145.6±15.3 (± SE) prey deliveries per nest were recorded in the Czech population and 167.4±19.7 prey deliveries per nest in the Finnish population. From the data recorded by the boxes, we calculated the number of prey a male delivered to the nest during each 30 min interval of a day.

### Prey species and mass delivered to boxes

We used two different methods to assess the diet composition: The pictures recorded by the camera allowed us to identify 77.6% of all prey delivered to the nest box (N = 3254) at least to the genus level. To support the accuracy of prey identification from the pictures, we recorded every 3–5 day all prey items in the nest boxes. We calculated the average weight of the prey delivered to the nest box by matching the weight of individuals trapped in the Czech population in 2004 and 2006, respectively from published long-term data from the Finnish population [26], [27]. For birds, we used the average weights given in the literature [28]. Prey items which we were unable to identify from the pictures were not used to calculate prey mass delivered to a nest box. Because prey delivered to the nest tend to be lighter than those captured in the field with snap-traps [29], using the weight of trapped individuals to estimate the weight of prey items males delivered to the nest box will underestimate the prey weight delivered to nests in the Czech population (see below).

### Statistical analyses

We used SAS 9.3 (SAS institute, Cary, North Carolina) to analyse our data. The snap-trapping data and the basic breeding data were analysed with either a General Linear Mixed Model (Normal distribution; identity-link: number of small mammals trapped, prey weight, date egg laying, fledging date), or with a Generalised Linear Mixed Model (Poisson distribution, log-link: clutch size, number of nestlings, number of fledglings, brood reduction). In all these analyses we included year as random effect to control for between-year differences.

For the analysis of the number of visits and the prey mass delivered within each 30 min bout we used a Generalised Linear Mixed Model in the GLIMMIX module. We used the Scaled Pearson statistics as parameter to assess the dispersion of our models [30]. Pearson statistics values around 1 indicate a good fit between the chosen error distribution and the error distribution of the data. The number of visits was best fitted with a Poisson distribution (log-link) (Scaled Pearson statistics = 0.84). Given that our data were overdispered (visual inspection of the Pearson residual plot), we added a multiplicative overdispersion parameter to the model (random _residual_ option in GLIMMIX, see [30]), which improved the fit of the model (Scaled Pearson statistics = 0.96). An overdispersion parameter only changes the variance-covariance matrix of the estimates by a certain factor, but does not alter any of the other parameter estimates [30]. For the analysis of the prey mass delivered each 30 min bout none of the distribution available in GLIMMIX provided a reasonable fit (best fit achieved with a Negative Binomial distribution: Scaled Pearson statistics = 3.76). Thus, we choose to transform the data (y = 1/(x+1)) and now a lognormal distribution (identity-link) fitted our data best when including a multiplicative overdispersion parameter to the model (Scaled Pearson statistics = 0.86).

We entered mean annual prey availability (based on snap trapping data, see above) sun angle, country, nestling requirements (number of nestlings multiplied by the age of nestlings) and night length (time between sunset and sunrise in min) as main effects into the model. In addition we also entered possible interactions of these into the model. We used box identity nested within country as random factor to control for the fact that we had repeated measures from the same box. To calculate the sun angle, we used a calculator available on the web (www.susdesign.com/sunangle/) and confirmed the accuracy of this tool by comparing with known sunrise and sunset times for our study sites.

We determined the best model using the corrected Akaike's information criterion (AICc; Table 2) [31]. Starting with the maximal model, we sequentially removed terms to construct all possible candidate models. To evaluate the fit of the different models, we calculated the ΔAICc which is the difference between the model with the lowest AICc in relation to the other models. In both analyses the best fitting model was much better than other models (ΔAICc≥2; Table 2; [31]), and thus we only give the detailed analyses of the best fitting models.

**Table 2:** Candidate model set of factors affecting the (a) number of prey items (Poisson distribution, log-link), and (b) the average prey mass delivered to nest within half-hour intervals (Lognormal distribution, identity-link).

a: number of prey items per 30 min			
factors	AICc	ΔAICc	wi
s+r+n+c+r*n+s*c+s*r+r*c+n*c+s*n+s*n*c	14384.93	0	0.74
s+r+n+c+r*n+s*c+r*c+n*c+s*n+s*n*c	14387.57	2.64	0.20
s+r+n+c+p+r*n+s*c+s*r+r*c+n*c+s*n+s*n*c	14390.1	5.17	0.06

b: number of prey items per 30 min			
factors	AICc	ΔAICc	wi
s+r+n+c+s*c+s*r+n*c+s*n+s*n*c	66604.71	0	0.662
s+r+n+c+s*c+s*r+n*c+s*n	66607.33	2.62	0.179
s+r+n+c+s*c+s*r+s*n	66607.67	2.96	0.151
s+r+n+c+s*c+s*r+r*c+n*c+s*n	66615.24	10.53	0.003
s+r+n+c+r*n+s*c+s*r+n*c+s*n	66615.52	10.81	0.003
s+r+n+c+p+s*c+s*r+n*c+s*n	66616.85	12.14	0.002
s+r+n+c+s*c+s*r+n*c	66618.69	13.98	0.001

s (sun angle), r (nestling requirements), n (night length), c (country), p (prey availability).

ΔAICc = difference in AICc relative to the best model; *wi* = ΔAICc weight of the model. All models with AICc weight (*wi*)≥0.001 are shown.

## Results

### Prey availability

The number of trapped small mammals, the main prey of Tengmalm's owl, did not differ between the Czech (mean ± SE = 3.8±1.7 items 100 trap nights, n_2004+2006_ = 6 squares) and the Finnish population (13.3±3.0 items/100 trap nights, n_2005_ = 8 squares; GLMM: country: F = 3.25, p = 0.1; including year as random factor). However, the species composition of trapped mammals differed between the two study sites. In the Czech site, the yellow-necked mouse *Apodemus flavicollis* (accounting for 71.3% of all trapped small mammals in 2004) and field voles *Microtus agrestis* (only species caught in 2006) were the dominant prey species. In the Finnish site, bank voles *Myodes glareolus* were the most abundant prey species (48.2%), while sibling voles *Microtus rossiaemeridionali*s (16.9%) and common shrews *Sorex araneus* (16.3%) were less abundant. Since these species differ in their body mass with shrews being much smaller than voles and mice, the average mass of prey was higher in the Czech population (mean weight ± SE = 23.3±0.7 g) than in the Finnish population (19.9±0.5 g; GLMM: F = 15.18, p = 0.0001; including year as random factor).

### Basic breeding data

Basic breeding data were collected from 39 nests in the Czech population (15 in 2004, 24 in 2006), and from 75 nests in the Finnish population (all in 2005; see Table 1).

In the Czech population, females initiated egg laying 2–3 weeks later than in the Finnish population, and consequently the fledging date was 2.5 weeks later in the Czech population (Table 1). The clutch sizes and number of hatched chicks were smaller in the Czech population than in the Finnish population (Table 1). Since birds breeding in the Finnish population had increased nestling loss through starvation (p = 0.0002; Table 1), the number of fledglings did not differ between the two populations (Table 1). In the Czech population 25 of the 39 nests (64.1%) were successful (i.e. at least one young fledged), and 55 of the 75 nests (73.3%) in the Finnish population were successful.

In the nests where we sampled the nestling provisioning rates (N = 12 nests in the Czech population; N = 9 nests in Finland) the laying date and fledgling date did not differ but clutch size was smaller in the Czech than in Finnish nests. Nevertheless, the numbers of young that fledged did not differ (Table 1). Thus, males in the Finnish population had to feed 1–2 young more than males in the Czech population before brood reduction occurred.

### Nestling provisioning

**Figure 1:**
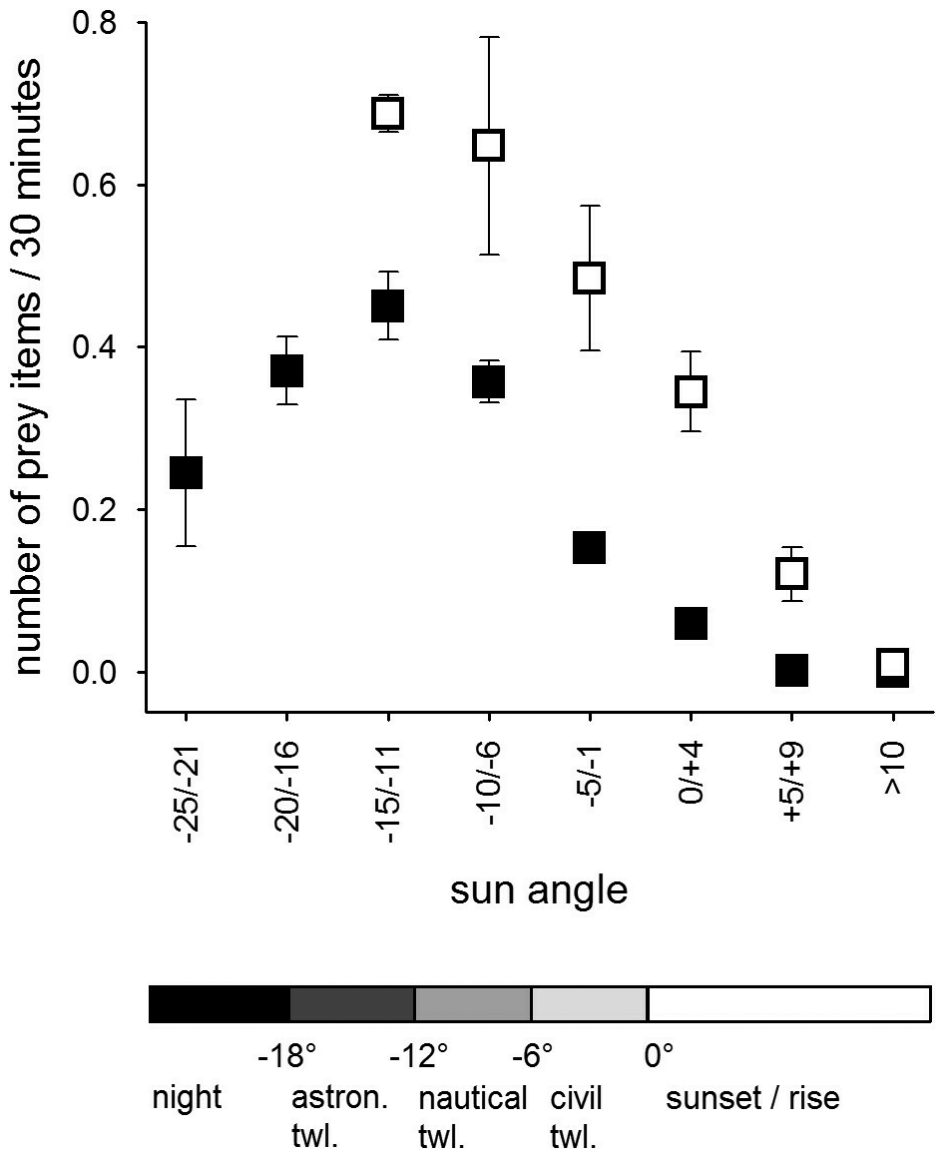
Mean (±SE) number of prey items delivered during 30 min periods in the Czech (filled squares) and the Finnish population (open squares) against the sun angle in relation to the horizon (in degrees, 0 = sunrise/sunset, twl. = twilight).

**Figure 2:**
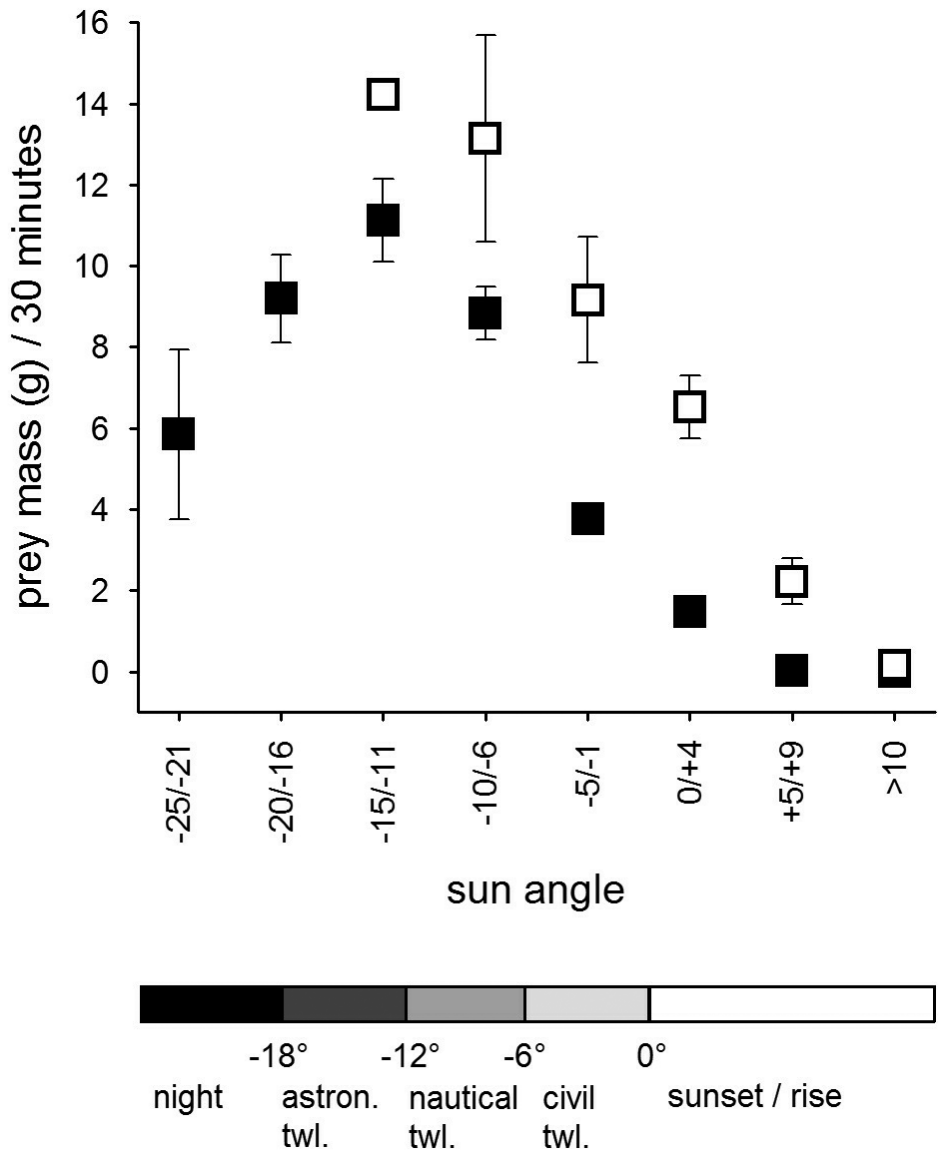
Mean prey mass (weight (g) ±SE) delivered during 30 min periods in the Czech (filled squares) and the Finnish population (open squares) against the sun angle in relation to the horizon (in degrees, 0 = sunrise/sunset, twl. = twilight).

**Figure 3:**
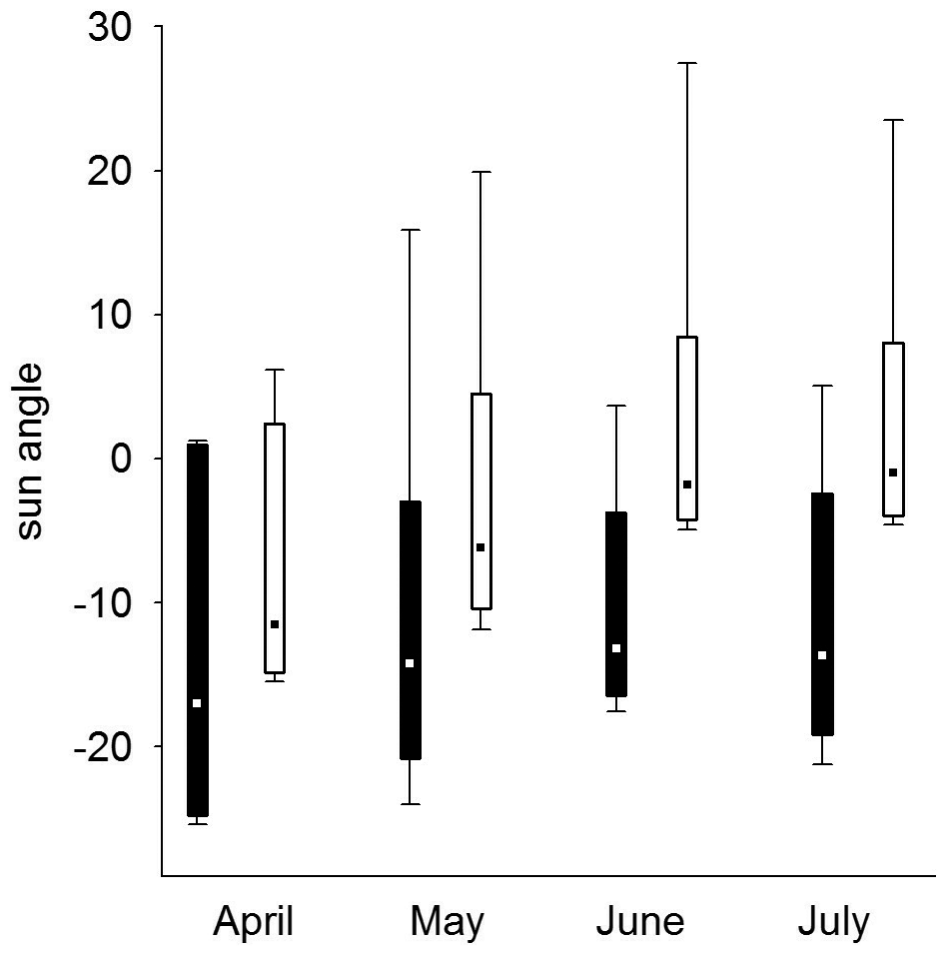
Change in hunting times for males throughout the breeding season in relation to the sun angle, based on prey delivery data in the Czech (filled squares) and Finnish population (open squares) (box: 5–95%, whiskers: non-outlier range, point: median).

**Figure 4:**
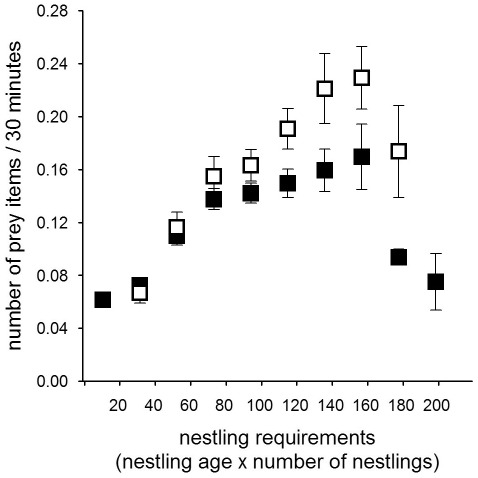
Mean (±SE) number of prey items delivered during 30 min periods in the Czech (filled squares) and Finnish population (open squares) in relation to the nestling requirement (nestling number×nestling age). The reduction of the feeding rate with older nestlings is a consequence of that broods fledge asynchronously.

**Figure 5:**
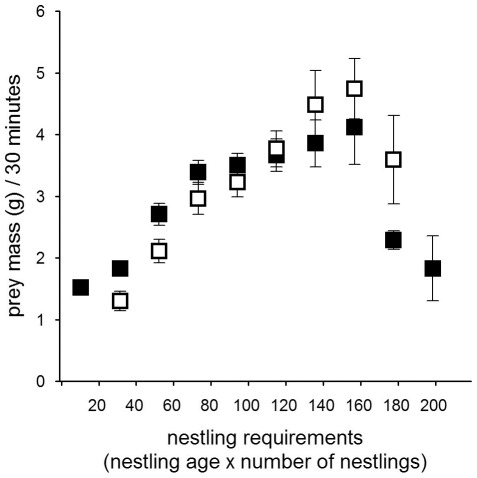
Mean prey mass (weight (g) ±SE) delivered during 30 min periods in the Czech (filled squares) and Finnish population (open squares) in relation to the nestling requirement (nestling number×nestling age). The reduction of the feeding rate with older nestlings is a consequence of that broods fledge asynchronously.

**Figure 6:**
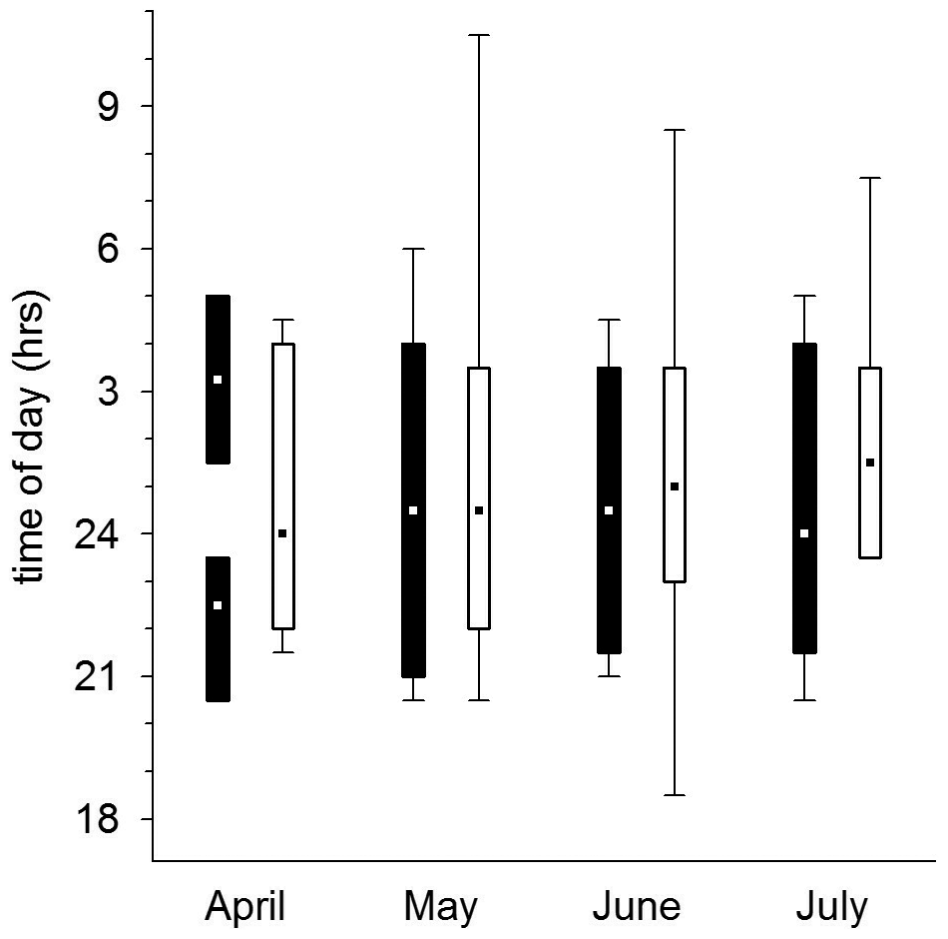
Change in hunting times of males in the Czech (filled squares) and Finnish population (open squares) (box: 5–95%, whiskers: non-outlier range, point: median), shown in relation to the months. Males in the Czech population have the fewest hours of hunting time in the beginning of the breeding season, whereas Finnish males have the fewest hours of hunting time by mid-summer, when most broods have large nestlings.

Males in the Czech population delivered on average 5.6±0.4 (mean ± SE) prey items per night, while males in the Finnish population delivered on average 7.8±1.0 prey items per night. Given the size difference in the available prey species between the two populations, males in the Czech population delivered larger prey than males in the Finnish population to their nests (Table 3). The prey delivery rate and the average prey mass males delivered to their nest during 30 min was influenced by the sun angle (i.e. light level), the night length, country as well as the nestling requirements (Table 2, 4, Fig. 1, 2, 3, 4, 5, 6).

**Table 3:** Number of prey items per nest and percentage of different prey items delivered by males to the nest-boxes in the Czech population (Ore Mountains) and the Finnish population (Kauhava region).

	Box identity	*Arvicolidae*	*Muridae*	*Gliridae*	*Soricidae*	Birds	Total number of prey items per nest	Mean weight prey
Czech population								
prey items delivered to individual nest (%)	302	41.5	51.2	0	4.4	2.9	205	24.4
	44	32.5	55	0	6.7	5.8	120	24.0
	74	40.3	49.6	2.3	3.9	3.9	129	24.5
	408	22.2	68.2	1.5	5.9	2.2	135	23.3
	409	29.5	63.8	1.3	2.7	2.7	149	24.1
	404	29.3	64.1	1.1	3.3	2.2	92	24.0
	406	60	0	0	16.7	23.3	30	25.7
	504	82	0	0	14.6	3.4	89	25.7
	541	74.2	0	0	19.3	6.5	93	25.1
	554	76.7	10.1	0	9.3	3.9	129	25.7
	577	52.9	1.4	1.4	15.7	28.6	70	26.0
	91	78.7	2.2	0	16.9	2.2	136	24.8
Finnish population								
prey items delivered to individual nest (%)	529	70.8	3.5	0	17.7	8	226	20.7
	692	73.1	1.9	0	16.7	8.3	108	21.1
	241	67.1	1.4	0	22.9	8.6	70	20.2
	887	69.4	1.6	0	21.5	7.5	186	20.3
	278	57.6	0	0	31.5	10.9	92	19.3
	527	63.6	2.3	0	25.8	8.3	132	19.6
	147	50.5	5.4	0	35.5	8.6	93	17.8
	936	58.2	5.5	0	28.6	7.7	91	18.8
	820	37.1	0.7	0	53.6	8.6	151	15.9

**Table 4:** Statistical analyses (Generalized Linear Mixed Models: GLIMMIX module in SAS 9.3) of factors affecting the (a) number of prey items (Poisson distribution, log-link), and (b) the average prey mass delivered to nest within half-hour intervals (Lognormal distribution, identity-link) in the Czech population (Ore Mountains; 12 nest boxes; number of days observed (mean ± SE): 25.8±2.2) and in the Finnish population (Kauhava region; 9 nest boxes; number of days observed: 22.9±2.4).

(a)						
Effect	Estimate	SE		Den DF	T-Value	P-Value
intercept	−0.17	0.27		24689	−0.61	0.55
box nested in country (random variable)	0.96	0.0009				
sun angle 1	−0.11	0.01		24689	−7.21	<0.0001
nestling requirements 2	0.003	0.003		24689	1.20	0.23
night length (min) 3	−8.76	1.26		24689	−6.97	<0.0001
country	−2.94	0.78	CZ<FIN	19	−3.76	0.001
nestl. requ.×night length	0.02	0.01		24689	1.58	0.11
sun angle×country	−0.28	0.05	CZ<FIN	24689	−5.57	<0.0001
sun angle×nestl. requ.	−0.000006	0.00006		24689	−0.10	0.92
sun angle×night length	−0.12	0.07		24689	−1.86	0.06
nestl. requ.×country	−0.005	0.001	CZ<FIN	24689	−3.47	0.0005
night length×country	9.42	2.37		24689	3.97	<0.0001
sun angle×night length×country	0.97	0.15	FIN<CZ	24689	6.24	<0.0001

(b)						
Effect	Estimate	SE		Den DF	T-Value	P-Value
intercept	−0.45	0.05		19	−9.45	<0.0001
box nested in country (random variable)	0.86	0.008				
sun angle 1	0.006	0.002		24691	3.3	0.001
nestling requirements 2	−0.003	0.0002		24691	−17.51	<0.0001
night length (min) 3	−0.06	0.19		24691	−0.34	0.73
country	−0.21	0.16	FIN<CZ	19	−1.32	0.20
sun angle×country	0.005	0.005	CZ<FIN	24691	0.94	0.35
sun angle×nestl. requ.	0.00008	0.000006		24691	13.27	<0.0001
sun angle×night length	0.04	0.007		24691	5.33	<0.0001
night length×country	1.01	0.48	CZ<FIN	24691	2.09	0.04
sun angle×night length×country	−0.05	0.01	FIN<CZ	24691	−3.02	0.003

Box identity nested within country was added as a random factor to the model to control for the repeated measurement at the same nest boxes.

1: sun angle (in degrees) in relation to the horizon. 0° = sunrise/sunset.

2: calculated as the product of number of nestling×age of oldest nestling.

3: duration of the night: minutes between sunset and sunrise.

Males delivered most food between dusk and dawn and had peak prey delivery rates (both number of prey and prey mass) at sun angles of −11° to −15° at both sites (Fig. 1, 2). In the Czech site prey delivery rates dropped during dark parts of the night (sun angles below −16°), while the nights at the Finnish site never were too dark for males to hunt. Over the breeding season, males at the Czech site showed only a small shift in their delivery peak (−17° in April to −14° in June and July), while males at the Finnish site moved their activity towards lighter parts of the night, shifting their peak of prey delivery from −11° in April to −1° in July (Fig. 3). Thus, during the period when males were feeding large young, males at the Finnish site delivered most prey with sun angles larger than 0° (Fig. 3).

Males increased their delivery rates (both the number of prey delivered and prey mass) with higher nestling requirements (Fig. 4, 5; Table 4), while the reduction of the prey delivery rate at the end of the nestling periods was a consequence of asynchronous fledging (Fig. 4, 5). As a consequence of the increased nestling requirements and the change in peak prey delivery rates over the season, the time available for hunting differed markedly between the populations (Fig. 6). Males in the Czech population seemed to be limited in their hunting time early on in the breeding season due to the darkness (very low sun angles) around midnight. Despite this constraint of darkness in the southern population, males in the Czech population had a substantially longer hunting time per night when feeding young (May: 420 min; June: 360 min; July: 390 min) than males in the Finnish population (May: 330 min; June: 270 min; July: 240 min; Fig. 6). Males in the Finnish population compensated for the shorter nights when feeding large young both by extending prey deliveries outside the dark period of the night and increasing the number of visits (Fig. 3; Table 4).

## Discussion

For diurnal birds, latitudinal differences in the reproductive effort are well established [5], [32]. Within species, individual reproductive investment increases with latitude, but the mechanism behind this pattern is still under debate [5]. It has been suggested that individuals breeding at low latitudes are either food constrained [9], [33], or time constrained leading to a lower reproductive investment than at higher latitudes [11]. Our results indicate that similar reproductive constraints may apply to nocturnal species, although in a reversed manner where individuals breeding at high latitudes are constrained in their foraging time during the reproductive period. To counteract this time constraint, male Tengmalm's owls extend prey deliveries more into the light period of the day, as well as by having higher prey delivery rates. However, since we only compare two populations we cannot draw more general conclusions, which will need further studies.

### Why do owls avoid hunting at daytime?

In contrast to diurnal species which have difficulties to forage in darkness [20], nocturnal hunters are capable to hunt at day time [13]. Still, despite that owls are powerful predators, Tengmalm's owls and other owl species avoid hunting at daytime and roost in cryptic locations [19], [34], which poses a limitation in particular for males in the Finnish population around midsummer. This behaviour might be a consequence of prey availability, hunting strategy, or increased predation risk at daytime. Many diurnal raptors successfully hunt small mammals at daytime, using quite similar hunting strategies as Tengmalm's owls [13], [35]. Moreover, Tengmalm's owl successfully hunt in Swedish Lapland around mid-summer when the sun is all day above the horizon [36]. Similarly, a late breeding male in the Finnish population successfully delivered in some instances prey items several hours after sunrise. Thus, it seems unlikely that prey behaviour or hunting strategy restrain the hunting time of Tengmalm's owls.

Rather, it seems likely that intra-guild predation [37] might be the reason why owls avoid hunting at day time. Tengmalm's owls and other small owl species have an increased risk of being killed by other predators [19], such as the diurnal goshawk *Accipiter gentilis* [13], [38], as well as the Ural owl *Strix uralensis* and eagle owl *Bubo bubo* [13], [39], both of which are also active during the light hours of the day [40], [41]. These predator species are relatively abundant at the Finnish study site but occur only rarely at the Czech site [42]. Despite this difference in predator abundance between the two sites, we found radio-tagged male owls killed by avian predators in both sites (Finland: 1 out of 27 tagged males killed [43]; Czech Republic 1 out of tagged 25 males killed; M. Zárybnická unpublished data). Thus Tengmalm's owl males seem to avoid to hunt during the period of the day when their own predation risk is high.

### Constraints of darkness

Despite that owls are adapted to hunt in the dark, their vision is still constrained by the darkest period of the night [20]. This appears to be a limiting factor for hunting males in the southern, Czech population because the number of prey deliveries was reduced at sun angles lower than 16° below the horizon (i.e. complete darkness). As mentioned above, neither an activity reduction of main prey in the middle of the night and during the day can explain these patterns, and many small mammals including voles of the genera *Microtus* and *Myode*s [44] are active all day and night.

### Why do owl females in Finland lay larger clutches?

Based on the differences in hunting time between the two populations, one would expect females in the Finnish population to lay smaller clutches than females in the Czech population, but this is not the case. However, the two populations not only differ in the daily time available for hunting, but also in prey availability. Voles in Northern Europe (including our Finnish study site) show multiannual cycles with peak years at three-year intervals [22], [45], creating very different breeding conditions from year to year. In contrast, voles in the Czech site do not undergo large changes in availability [24], but mice show irregular low peaks in beech mast years [46]. While owl broods in the Finnish population suffered from higher brood reduction rates than in the Czech population, females in the Finnish population are partly able to adjust the number of eggs according to the food availability [17]. Nevertheless, given the higher and partly unpredictable variation in food availability, Finnish owl females lay in some years too many eggs in relation to the food availability. Consequently, the brood reduction rates between 2000 and 2009 in the two populations were consistently higher in the Finnish than in the Czech population under both increasing and decreasing availability of their main prey during the breeding season (GLIMMIX: Poisson error distribution, prey abundance * study population: F = 13.20, p = 0.0004; including year as random factor; M. Zárybnická & E. Korpimäki unpublished data). Brood reduction which is facilitated by the high degree of hatching asynchrony of Tengmalm's owls [47] acts as a mechanism which compensates for the incomplete information regarding prey availability Finnish females have during egg-laying [48]. This suggests that different selective pressures act upon life-history decisions in environments with relatively stable and predictable and relatively unstable and unpredictable food availability [49].

To conclude, our between-population comparison supports the notion that reproductive patterns vary across latitudes. In day-active species latitudinal variation in reproductive investment has been suggested to be a result of food availability and energetic constraints [11], [33]. Our results suggest that nocturnal species in addition to food availability also are limited by the darkness (at lower latitudes) and predation risk, and the latter factor has been shown to affect foraging patterns and consequently reproductive investment in other species as well [50], [51]. In particular, the unpredictable and unstable food availability might induce females in the Finnish population to lay larger clutches. This in turn might result in high rates of brood reduction in years with decreasing availability of main prey in summer, but a higher reproductive output in years with increasing and high availability of main prey in summer.

## Supporting Information

Appendix S1 Mean monthly temperature, total monthly rainfall and maximum monthly snow cover in the Czech Republic (Ore Mountains) and in Finland (Kauhava region) in 2000–2009.
(DOCX)
