# Supplementary material for: Dark or Short Nights: Differential Latitudinal Constraints in Nestling Provisioning Patterns of a Nocturnally Hunting Bird Species
Source: PLoS One. 2012 May 16;7(5):e36932. doi: 10.1371/journal.pone.0036932 (PMC3353992; doi:10.1371/journal.pone.0036932)
Supplement: Appendix S1 — Mean monthly temperature, total monthly rainfall and maximum monthly snow cover in the Czech Republic (Ore Mountains) and in Finland (Kauhava region) in 2000–2009. (DOCX) [file pone.0036932.s001.docx]

|  | Czech Republic (Ore Mts.) | | | | | Finland (Kauhava region) | | | | |
| --- | --- | --- | --- | --- | --- | --- | --- | --- | --- | --- |
|  | March | April | May | June | July | March | April | May | June | July |
| temperature (°C) | 0.1 | 5.4 | 10.1 | 12.8 | 14.4 | 3.7 | 3.8 | 9.5 | 13.9 | 16.9 |
| SE | 0.4 | 0.6 | 0.4 | 0.5 | 0.5 | 1.0 | 0.3 | 0.3 | 0.3 | 0.4 |
| rainfall (mm) | 100.3 | 49.3 | 91.3 | 82.5 | 106.8 | 21.5 | 26.3 | 46.9 | 51.4 | 72.9 |
| SE | 21.1 | 11.1 | 12.9 | 6.7 | 11 | 3.1 | 6.5 | 6 | 5 | 9.6 |
| snow cover (cm) | 47.2 | 9.1 | 0.3 | 0 | 0 | 18.9 | 2.7 | 0 | 0 | 0 |
| SE | 11.8 | 3.9 | 0.3 | 0 | 0 | 6.2 | 2.6 | 0 | 0 | 0 |
